# Trends in opioid and non-opioid treatment for chronic non-cancer pain and cancer pain among privately insured adults in the United States, 2012–2019

**DOI:** 10.1371/journal.pone.0272142

**Published:** 2022-08-10

**Authors:** Sachini Bandara, Mark C. Bicket, Emma E. McGinty

**Affiliations:** 1 Department of Mental Health, Johns Hopkins Bloomberg School of Public Health, Baltimore, Maryland, United States of America; 2 Center for Mental Health and Addiction Policy Research, Johns Hopkins Bloomberg School of Public Health, Baltimore, Maryland, United States of America; 3 Department of Health Policy and Management, Johns Hopkins Bloomberg School of Public Health, Baltimore, Maryland, United States of America; 4 Department of Anesthesiology, University of Michigan School of Medicine, Ann Arbor, Michigan, United States of America; 5 Michigan Opioid Prescribing Engagement Network, University of Michigan, Ann Arbor, Michigan, United States of America; PLoS ONE, UNITED STATES

## Abstract

Recent clinical guidelines have emphasized non-opioid treatments in lieu of prescription opioids for chronic non-cancer pain, exempting cancer patients from these recommendations. In this study, we determine trends in opioid and non-opioid treatment among privately insured adults with chronic non-cancer pain (CNCP) or cancer. Using administrative claims data from IBM MarketScan Research Databases, we identified privately-insured adults who were continuously enrolled in insurance for at least one calendar year from 2012 to 2019. We identified individuals with CNCP diagnosis, defined as a diagnosis of arthritis, headache, low back pain, and/or neuropathic pain, and a individuals with cancer diagnosis in a calendar year. Outcomes included receipt of any opioid, non-opioid medication, or non-pharmacologic CNCP therapy and opioid prescribing volume, MME-per-day, and days’ supply. Estimates were regression-adjusted for age, sex, and region. Between 2012 and 2019, the proportion of patients who received any opioid decreased across both groups (CNCP: 49.7 to 30.5%, p<0.01; cancer: 86.0 to 78.7%, p<0.01). Non-opioid pain medication receipt remained steady for individuals with CNCP (66.7 to 66.4%, p<0.01) and increased for individuals with cancer (74.4 to 78.8%, p<0.01), while non-pharmacologic therapy use rose among individuals with CNCP (62.4 to 66.1%, p<0.01). Among those prescribed opioids, there was a decrease in the receipt of at least one prescription with >90 MME/day (CNCP: 13.9% in 2012 to 4.9% in 2019, p<0.01; Cancer: 26.2% to 7.6%, p<0.01); >7 days of supply (CNCP: 56.3% to 30.7%, p <0.01; Cancer: 47.5% to 22.7%, p<0.01), the mean number of opioid prescriptions (CNCP: 5.2 to 3.9, p<0.01; Cancer: 4.0 to 2.7, p<0.01) and mean MME/day (CNCP: 49.9 to 38.0, p<0.01; Cancer: 62.4 to 44.7, p<0.01). Overall, from 2012–2019, opioid prescribing declined for CNCP and cancer, with larger reductions for patients with CNCP. For both groups, reductions in prescribed opioids outpaced increases in non-opioid alternatives.

## Introduction

Over the past decade, the federal government, state governments, medical societies, health systems and insurers have issued clinical guidelines and policies designed to reduce opioid prescribing [1,2]. Higher doses, longer days supply, and long-acting prescription opioid formulations are associated with increased risk of long-term opioid use and overdose [3–5]. Co-prescribing of benzodiazepine is also associated with increased risk of fatal overdose [6,7]. Some patients taking long term opioid treatment can also have higher risk for increased sensitivity to pain [8]. A commonly cited concern by pain experts, advocates, and patients is that efforts to reduce opioid prescribing may lead to inadequate management of chronic non-cancer pain (CNCP) and cancer-related pain [1,2,9–15]. Overall U.S. opioid prescribing rates have decreased from 81.2 prescriptions per 100 people in 2010 to 43.3 prescriptions per 100 people in 2020 [16]. Data on trends for the population of patients with CNCP and cancer are needed to understand how treatment of these types of pain has shifted during an era of more restricted opioid prescribing.

Pain-related conditions associated with CNCP where opioids were historically considered acceptable first-line treatment and frequently prescribed include low back pain, arthritis, serious headache and neuropathic pain [17]. In 2016, the Centers for Disease Control and Prevention (CDC) released a guideline on opioid prescribing for CNCP, which recommended that non-opioid pharmacologic and non-pharmacologic therapies be used as first-line treatments [1]. The CDC guideline recommended that when opioids are prescribed, prescriptions should be short in duration, have the lowest effective dose, and be combined with non-opioid therapies as appropriate. These CDC recommendations are consistent with other opioid prescribing guidelines, all of which specifically exempt individuals in active cancer treatment from recommended limits on opioid prescribing [1,18–22]. In February 2022, the CDC released updated draft guidance softening recommendations for ceilings on opioid dosage, instead favoring clinical judgement, but still recommending non-opioid treatments as first line therapy for CNCP [23].

Trends in opioid prescribing and delivery of non-opioid therapies among CNCP patients compared to cancer patients over the past decade are not well understood. Extant research on opioid prescribing patterns among CNCP patients has documented increases in opioid prescribing prior to 2010 using national samples and decreases in opioid prescribing after 2010 among non-national samples and populations with specific conditions, like arthritis [24–27]. Prior research has documented reduced opioid prescribing by oncologists among Medicare Part D beneficiaries [28] and a rise in opioid-related hospitalizations among patients with cancer [29]. No studies using national samples of adults have examined trends in receipt of opioid and non-opioid therapies among CNCP patients versus cancer patients in recent years. This study builds upon existing research by examining treatment trends among a national sample of privately insured adults experiencing one or more of four common CNCP conditions or a cancer diagnosis from 2012 to 2019.

## Materials and methods

### Sample

We conducted a retrospective cohort study using the IBM MarketScan Research Databases from 2012 to 2019, which included health insurance claims and encounters for privately insured employees and their dependents from approximately 100 insurance companies in the US covering 26.1 to 53.1 million enrollees, depending on the year.

The sample included adults aged 18 years and older who were continuously enrolled in a self-insured employer-sponsored insurance plan for at least one calendar year from January 2012 through December 2019. In every year, we identified individuals enrolled for the entire year with CNCP diagnoses in that year, defined as those individuals with at least one inpatient or two outpatient diagnoses of low backpain, neuropathic pain, serious headache or arthritis (e.g. osteoarthritis and rheumatoid arthritis), using International Classification of Diseases, Ninth Revision (ICD-9) and 10^th^ Revision (ICD-10) codes (Codes in S1 Appendix). These conditions were chosen because they are among the most common pain conditions, could be well-identified in claims data, and had historically been treated with opioids as the recommended first line therapy prior recent guideline modifications [17]. Individuals were considered to have CNCP only in calendar years in which they met these criteria, due to fluctuations in pain symptoms over time for many chronic pain conditions [30–33]. We also identified individuals with a cancer diagnosis in each calendar year, excluding skin cancer diagnoses. ICD-9 and ICD-10 cancer diagnosis codes were identified via the National Cancer Institute, Surveillance, Epidemiology and End Results Program Casefinding Lists [34]. We excluded individuals with both cancer and CNCP diagnoses in the same calendar year.

### Outcomes

For both groups, we identified whether individuals received at least one opioid prescription, at least one clinical guideline-concordant non-opioid prescription pain medication, and, among individuals who were prescribed an opioid, the number of opioid prescriptions, duration, and strength of each prescription. For individuals with CNCP, we also identified if they received at least one guideline-concordant CNCP non-pharmacologic therapy.

We identified opioid prescriptions using the 2018 CDC Opioid NDC and Oral MME Conversion File [35]. We included all forms of opioids (i.e. tablet, suspension, etc.) long and short-acting opioids and excluded buprenorphine formulations that are primarily used to treat opioid use disorder. We also identified clinical guideline-concordant non-opioid pain medication prescriptions used for treatment of chronic pain and non-pharmacologic therapies used to treat chronic pain. National drug codes for medications and procedure codes for non-pharmacologic CNCP therapies were compiled by our study team based on existing clinical guidelines, as outlined in a previously published study protocol (S1 Appendix) [36]. We classified every non-opioid medication into the following categories: 5HT1 agonists, anticonvulsants, local anesthetic, NSAIDS, SNRIs, SSRIs, tricyclic antidepressants, skeletal muscle relaxants, steroids or other. The other category included prescriptions such as beta-blocking agents, acetaminophen combination products, ketamine, caffeine and other non-opioid prescriptions. We classified each non-pharmacologic therapy as surgical, minimally invasive or non-invasive.

For every individual that was prescribed an opioid, we also examined the number of opioid prescriptions, duration and strength of each prescription. The duration of the prescription was measured as the number of days of supply. To allow for comparison of strength across opioids of different types, we calculated the morphine milligrams equivalent (MME) by multiplying days of supply, dose and MME conversion factor. The dose and MME conversion factor for each drug was linked to Marketscan data from the 2018 CDC Opioid NDC and Oral MME Conversion File [35]. We calculated whether or not an individual received at least one prescription >90 MME-per-day (MME/day) or >200 MME/day and whether they received at least one prescription with >7 days of supply and >30 days of supply. These cutoffs were chosen because 2016 CDC guidance noted them as being associated with increased likelihood of misuse and recommended avoiding administering prescriptions exceeding these cut-points [1]. We calculated the average MME/day across all prescriptions per person, which was calculated as the sum of MME across all prescriptions divided by the total number of days in the calendar year a person was prescribed an opioid (i.e. taking the sum of days of supply across all prescriptions and then subtracting the days where multiple opioid prescriptions overlapped as to not double count these days, a technique that has been used in prior opioid prescribing research) [5,37].

### Primary analyses

We calculated regression-adjusted estimates of all outcomes separately for each group for each calendar year. Estimates were adjusted for age, sex, and region of residence. Standard errors were clustered at the individual level, and Wald tests were used to test for differences between estimates in a given year relative to the prior year and differences between 2012 and 2019 estimates.

### Secondary analysis

Given that clinical guidelines changed and began recommending non-opioid pain medications and non-pharmacologic therapies in place of opioids as first-line CNCP treatment during our study period, we conducted a secondary analysis assessing substitution of clinical guideline-concordant non-opioid therapies in place of opioid prescriptions among individuals with CNCP. We conducted this analysis among the subset of the sample with CNCP who were prescribed an opioid in the previous year. Among those individuals, we calculated the percentage of individuals who experienced reduced opioid prescribing. We defined reduced opioid prescribing using one of two benchmarks: 1) individuals whose yearly average MME/day reduced by at least 20 MME/day compared to the prior year or 2) individuals who received a lesser number of opioid prescriptions compared to the prior year. The 20 MME/day cutoff was chosen because CDC guidelines cited several studies that found increased overdose risk associated with higher opioid dosage comparing individuals with < 20 MME/day with higher dosage categories. Among CNCP patients with reduced opioid prescribing, we measured non-opioid substitution in four ways: 1) the percentage of CNCP patients who had an increase in the number of non-opioid pain medication prescriptions, but no increase in non-pharmacologic therapies, compared to the prior year; 2) the percentage of CNCP patients who had an increase in the number of non-pharmacologic therapies, but no increase in non-opioid pain medication prescriptions, compared to the prior year; 3) the percentage of CNCP patients who had increase in both the number of non-opioid prescriptions and the number of non-pharmacologic therapies compared to the prior year; and 4) no non-opioid substitution (i.e., none of the above). We calculated unadjusted descriptive statistics of these percentages and chi-2 tests to test for differences across years.

## Results

### Sample characteristics

The number of individuals with CNCP in the sample ranged from 1,717,340 to 2,220,248 across years. (Data for 2012 and 2019 in Table 1, data for all years in S2 Appendix) The number of individuals with cancer ranged from 7,710 to 13,687 across years. We excluded between 3,302 and 7,293 patients with both CNCP and cancer depending on the calendar year. Across years and diagnoses groups, the yearly mean age ranged from 46 to 53, and the percentage of female patients ranged from 48 to 65%. Thirteen to 22% of participants were from the Northeast region across all years and diagnosis groups, 22–26% from the North Central region, 37–47% from the South region, and 14–23% from the West region. Among those with CNCP, the percentage of individuals with each CNCP diagnoses ranged from 54–58% for low backpain, 29–31% for serious headache, 28–29% for arthritis, and 12–16% for neuropathic pain across years. The percentage of individuals with more than one type of CNCP diagnosis ranged from 23–26% across years.

**Table 1:** Characteristics of individuals with chronic non-cancer pain (CNCP) or cancer, 2012–2019.

	Chronic Non-Cancer Pain		Cancer	
	2012 (1,717,340)	2019 (1,556,737)	2012 (8,509)	2019 (8,515)
Mean Age	46	46	51	53
			
Female, % (n)	62	61	65	48
	(1,072,140)	(954,234)	(5,515)	(4,048)
Region, % (n)				
Northeast	13	13	17	17
	(223,665)	(202,227)	(1,404)	(1,447)
North Central	23	26	24	24
	(392,697)	(396,943)	(2,020)	(2,039)
South	41	47	38	45
	(710,601)	(734,646)	(3,218)	(3,809)
West	23	14	22	14
	(387,545)	(218,669)	(1,852)	(1,186)
Unknown	< 1	< 1	< 1	<1
	(2,832)	(4,252)	(15)	(34)
CNCP Diagnosis, % (n)				
Low Back Pain	54	58		
	(926,564)	(895,649)		
Serious Headache	29	31		
	(491,935)	(483,516)		
Arthritis	28	29		
	(480,408)	(453,026)		
Neuropathic Pain	15	12		
	(265,330)	(184,077)		
> 1 CNCP Diagnosis, % (n)	23	25		
	(388,687)	(393,295)		

Note: Data Source: IBM MarketScan Research Databases 2012–2019. Sample included adults aged 18 years and older who were continuously enrolled in a self-insured employer-sponsored insurance plan for at least one calendar year. CNCP diagnoses defined as those individuals with at least one inpatient or two outpatient diagnoses of low backpain, neuropathic pain, serious headache or arthritis.

### Opioid and non-opioid treatment trends

**Fig 1:**
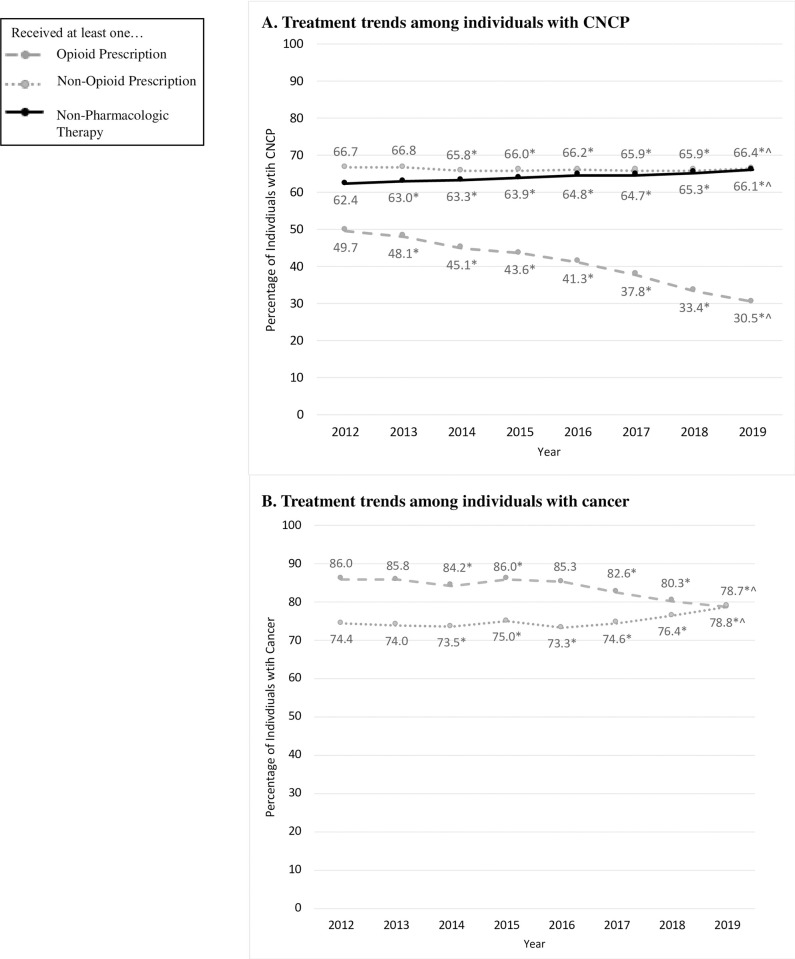
Adjusted opioid and non-opioid treatments among individuals with chronic non-cancer pain (CNCP) or cancer, 2012–2019.

The adjusted percentage of individuals with CNCP diagnoses prescribed an opioid decreased 19.2 percentage points (49.7% to 30.5%, p<0.01) from 2012 to 2019 (Fig 1). The adjusted percentage of this group prescribed a clinical guideline-concordant non-opioid pain medication remained steady (66.7% to 66.4%, p<0.01) from 2012 to 2019. The adjusted percentage of individuals with CNCP receiving a guideline-concordant non-pharmacological therapy increased 3.7 percentage points from 2012 to 2019 (62.4% to 66.1%, p<0.01).

The adjusted percentage of the group with cancer diagnoses prescribed an opioid decreased 7.3 percentage points from 2012 to 2019 (86.0% to 78.7%, p<0.01). The adjusted percentage of this group prescribed a clinical guideline-concordant non-opioid pain medication increased 4.4 percentage points from 2012 to 2019 (74.4% to 78.8%, p<0.01).

The most commonly prescribed clinical guideline-concordant non-opioid pain medications among individuals with CNCP diagnoses were antidepressants (ranging from 23.3–23.7% of all non-opioid prescriptions and comprised of 6.5–6.7% SNRI, 13.6–14.0% SSRI, and 2.8–3.2% TCA across all years), NSAIDs (ranging from 16.6–17.7% across all years) and anticonvulsants (15.8–16.4% across all years) (S3 Table 1 in S3 Appendix). The most commonly prescribed clinical guideline-concordant non-opioid pain medications among individuals with cancer diagnoses were antidepressants (ranging from 17.7–23.4% of all non-opioid prescriptions, comprised of 4.0–5.9% SNRI, 12.4–16.0% SSRI, and 1.0–1.8% TCA across all years), steroids (ranging from 17.4–19.4%) and anticonvulsants (ranging from 13.8–19.5%).

Among patients with CNCP, the percentage of patients who received at least one guideline concordant non-invasive procedure ranged from 42.4 to 50.9% across all years, the percentage who received at least one guideline concordant surgical procedure ranged from 36.4 to 30.8% and the percentage who received at least one guideline concordant minimally invasive procedure ranged from 22.6 and 23.7% (S3 Table 2 in S3 Appendix).

### Opioid prescription characteristics

Among individuals who were prescribed an opioid, the adjusted mean number of opioid prescriptions per person decreased from 5.2 to 3.9 (p<0.01) in the CNCP sample and 4.0 to 2.7 (p<0.01) in the cancer sample from 2012 to 2019 (Table 2). From 2012 to 2019, the adjusted mean MME/day per person decreased from 49.9 to 38.0 (p<0.01) in the CNCP sample and 62.4 to 44.7 (p<0.01) in the cancer sample. The adjusted mean number of days a year with an opioid prescription also decreased from 2012 to 2019 from 74.2 to 59.6 days per person in the CNCP sample (p<0.01) and 34.8 to 22.3 days (p<0.01) in the cancer sample.

**Table 2:** Opioid prescription characteristics among individuals with chronic non-cancer pain or cancer who received an opioid prescription, 2012–2019.

	Chronic Non-Cancer Pain		Cancer	
	2012	2019	2012	2019
Number of opioid prescriptions per person, mean [95%CI]	5.2	3.9^	4.0	2.7^
	[5.2, 5.3]	[3.9, 3.9]	[3.9, 4.1]	[2.6, 2.8]
MME/day among opioid prescriptions per person, mean [95%CI]	49.9	38.0^	62.4	44.7^
	[49.7, 50.1]	[37.9, 38.1]	[60.9, 63.8]	[43.3, 46.1]
Number of days per year with opioid prescriptions person, mean [95%CI]	74.2	59.6^	34.8	22.3^
	[74.0, 74.5]	[59.3, 59.9]	[33.4, 36.1]	[21.1, 23.5]
Percentage of individuals w/ a prescription…				
>90 MME/day [95%CI]	13.9	4.9^	26.2	7.6^
	[13.9, 14.0]	[4.9, 5.0]	[25.2, 27.2]	[7.0, 8.3]
>200 MME/day [95%CI]	2.5	0.8^	3.6	1.4^
	[2.5, 2.5]	[0.8, 0.9]	[3.2, 4.1]	[1.1, 1.7]
>7 Days Supply [95%CI]	56.3	30.7^	47.5	22.7^
	[56.2, 56.4]	[30.6, 30.9]	[46.3, 48.6]	[21.8, 23.7]
>30 Days Supply [95%CI]	3.6	0.9^	1.2	0.3^
	[3.6, 3.7]	[0.9, 0.9]	[0.9, 1.4]	[0.2, 0.4]

Note: Data Source: IBM MarketScan Research Databases 2012–2019. Estimates adjusted for sex, age, region of residence. ^ denotes Wald test for differences between 2012 and 2019 p <0.05.

Among those who were prescribed an opioid, the percent of individuals who received at least one opioid prescription with >90 MME/day dropped 9 percentage points (13.9 to 4.9%, p<0.01) in the CNCP sample and 18.6 percentage points (26.2 to 7.6%, p<0.01) in the cancer sample. Among those who were prescribed an opioid, the percent of individuals who received at least one opioid prescription with >200 MME/day dropped 1.7 percentage points (2.5 to 0.8%, p<0.01) in the CNCP sample and 2.2 percentage points (3.6 to 1.4%, p<0.01) in the cancer sample.

Among those who were prescribed an opioid, the percent of individuals who received at least one opioid prescription with >7 days of supply dropped 25.6 percentage points (56.3 to 30.7%, p<0.01) in the CNCP sample and 24.8 percentage points (47.5 to 22.7%, p<0.01) in the cancer sample. Among those who were prescribed an opioid, the percent of individuals who received at least one opioid prescription with >30 days of supply dropped 2.7 percentage points (3.6 to 0.9%, p<0.01) in the CNCP sample and 0.9 percentage points (1.2 to 0.3%, p<0.01) in the cancer sample. The unadjusted values of all outcomes (S4 Appendix) and sensitivity analyses using a sample with both CNCP and cancer diagnoses (S5 Appendix) followed similar trends.

### Secondary analysis

**Fig 2:**
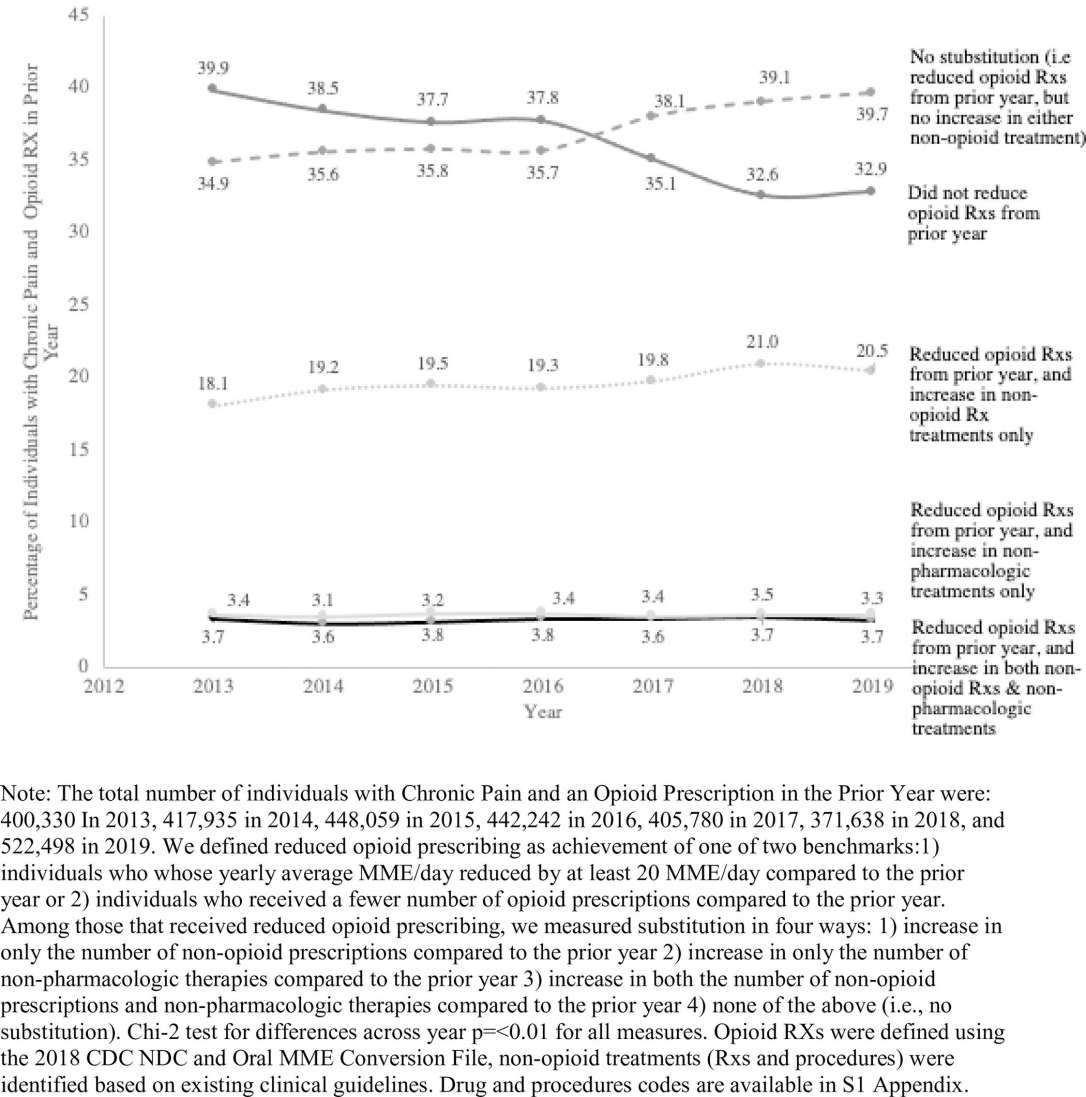
Year-to-year within-patient changes in chronic non-cancer pain treatment among patients with opioid prescriptions in the prior year.

Among individuals with CNCP, the percentage of individuals that did not experience reduced opioid prescribing compared to the prior year decreased over time from 39.9% in 2013 to 32.9% in 2019 (p<0.001), meaning that the percentage that did experience reduced opioid prescribing increased over time from 60.1% in 2013 to 67.1% in 2019 (p<0.001) (Fig 2). We examined substitution, which was defined as experiencing reduced opioid prescribing and an increase in either guideline-concordant non-opioid prescriptions or non-pharmacologic therapy and found that substitution with non-opioid prescriptions was greater than substitution with non-pharmacologic treatments in 2013, 18.1% of CNCP patients substituted opioids with non-opioid prescriptions only, relative to 21.0% in 2019 (p<0.001). Opioid substitution with non-pharmacologic therapies only occurred among 3.4% of CNCP patients in 2013 and 3.5% of CNCP patients in 2019 (p<0.0001). The proportion of CNCP patients who substituted reduced opioids with both non-opioid pain medications and non-pharmacologic therapies was unchanged from 2013 to 2019 (3.7%).

## Discussion

In a national sample of privately insured adults, opioid prescribing for individuals with CNCP decreased from 2012 to 2019, but delivery of guideline-concordant non-opioid CNCP treatments recommended by clinical guidelines [1,18–22] introduced during the study period did not increase in similar magnitudes. Compared to prior research on opioid prescribing trends in the overall U.S. population, prevalence of receipt of opioid prescriptions was higher in this privately-insured CNCP population and declines in opioid prescribing over time were greater [38,39]. Within individual patients with CNCP, roughly a third of individuals experienced no reduction in opioid prescribing compared to the prior year, another third had reductions in opioid prescribing compared to the prior year but no corresponding increase in non-opioid treatments, and roughly a third experienced reduced opioid prescribing and substitution of non-opioid treatments. Among those that experienced substitution, individuals largely experienced increases in non-opioid prescriptions. This suggests that the increases in non-pharmacologic treatment over time observed in the overall sample are being driven by increased delivery among patients who have not recently received an opioid prescription, as opposed to substitution of non-pharmacologic therapies among those currently using opioids to manage their CNCP. This may be in part due to inconsistent insurance coverage and utilization management policies for non-pharmacologic CNCP therapies [40].

While clinical guidelines continue to endorse prescription opioid treatment for cancer-related pain, declines in receipt of any opioid prescription were observed for people with cancer diagnoses, though these declines were smaller in magnitude than those observed among people with CNCP diagnoses. Future research is needed to understand the clinical implications of this decline, as well as observed decreases in MME/day and days’ supply of opioid prescriptions, among cancer patients. On the one hand, this finding may signal a cooling effect on opioid prescribing that could contribute to poorly controlled cancer pain. On the other hand, this finding may suggest reductions in high-risk opioid prescribing practices, such as high dosage prescribing, which are shown to contribute to increased risk of opioid overdose death among cancer patients [41].

One potential unintended consequence of efforts to limit opioid prescribing is that providers may engage in “gaming” behavior and provide patients with multiple prescriptions, larger doses, or greater days of supply to avoid callbacks for refills [42]. Therefore, examining the number of prescriptions, MME and days of supply can provide further insight. Among both CNCP and cancer patients, we find decreases in the average number of opioid prescriptions, MME/day and total number of days per year with an opioid prescription from 2012–2019, which is not consistent with gaming behavior.

Our findings align with prior research suggesting that receipt of opioid prescriptions among the general population decreased following the release of the CDC guidelines in 2016 [43]. However, our results do not suggest a clear CDC guideline effect, as the declines observed started prior to 2016. Other clinical guidelines were also released before and after 2016 that recommended decreased prescribing of opioids for CNCP, and may account for some of the observed trend [18–22]. Study results provide insight into pain treatment trends at a time of active opioid prescribing policy changes at the federal, state, insurer, and health-system levels, for example by 2019 33 states had policies requiring providers to check their state’s prescription drug monitoring program (PDMP) prior to prescribing an opioid and 35 states had policies limiting the dose and/or duration of opioid prescriptions, many laws enacted in the last decade [44]. While some policies explicitly exempt opioid prescribing for CNCP as well as for cancer–for example, most state opioid prescribing limit laws target opioid prescribing for acute pain [45]–prior research suggests that law implementation may, in practice, influence all opioid prescriptions rather than the subset for acute pain [44]. However more recent literature suggests that the effects of prescribing laws on chronic pain prescribing at the state-level are limited [46].

These results need to be taken in the context of several limitations. First, because our dataset was limited to administrative claims data. Therefore, we cannot determine the socioeconomic status of individuals in the sample, the clinical appropriateness of therapies, the consumption of opioids illegally or not paid for by insurance, or key clinical outcomes such as pain relief. Administrative claims data may also have data entry errors and misclassification, however to mitigate this we used measures of chronic non-cancer pain, cancer and therapies used in other claims analyses and guided by clinical guidelines. Second, our analytic sample included adults who were continuously enrolled in a self-insured employer-sponsored insurance plan for at least a full calendar year. Generalizability to other populations, such as Medicare and Medicaid beneficiaries and those without continuous insurance enrollment may be limited. Of particular note, these inclusion criteria led to a relatively small sample size of people with cancer diagnoses (18,700 in our sample, out of an estimated 18.1 million adults diagnosed with cancer, excluding skin cancer, in 2018 in the U.S.) Third, opioid prescribing in the general population varies widely by prescriber and geography, which was not examined in this study [47–49]. Fourth, while we measure receipt of an opioid prescription, this does not measure actual opioid consumption. Fifth, we did not include fibromyalgia as a CNCP condition, because there was a significant decrease in the number of fibromyalgia patients identified starting in 2015 that was attributed to the shift from ICD-9 to ICD-10 diagnosis codes.

## Conclusions

From 2012–2019, individuals with CNCP and individuals with cancer have experienced declines in opioid prescribing without corresponding increases in receipt of non-opioid therapies. Further investigation is needed to examine how these changes influence the management of pain among patients with CNCP and/or cancer.

## Supporting information

S1 Appendix Diagnosis and procedure codes. (PDF)

S2 Appendix Yearly results. (PDF)

S3 Appendix Non-opioid medication and non-pharmacologic procedure types. (PDF)

S4 Appendix Unadjusted results. (PDF)

S5 Appendix Results for sample with chronic non-cancer pain and cancer. (PDF)
