# Supplementary material for: Trends in opioid and non-opioid treatment for chronic non-cancer pain and cancer pain among privately insured adults in the United States, 2012–2019
Source: PLoS One. 2022 Aug 10;17(8):e0272142. doi: 10.1371/journal.pone.0272142 (PMC9365134; doi:10.1371/journal.pone.0272142)
Supplement: S1 Appendix — (PDF) [file pone.0272142.s001.pdf]

S1 Appendix. Diagnosis and Procedure Codes

Chronic Pain Diagnosis Codes

**Low Back Pain**						
ICD-9	721.3	721.42	722.52	722.73	724.02	724.03
	724.2	724.3	724.4	724.5		
ICD-10	M43.06	M43.07	M43.16	M43.17	M47.16	M47.26
	M47.27	M47.816	M47.817	M47.896	M47.897	M48.06*
	M48.07	M51.06	M51.16	M51.17	M51.26	M51.27
	M51.36	M51.37	M54.16	M54.17	M54.3*	M54.4*
	M54.5	M54.89	M54.9	S39.012$	S39.023$	S39.092$
**Headache**						
ICD-9	307.81	339.*	346.*	349	723.8	784
ICD-10	G43.*	G44.*	G97.1	M54.81	R51	
**Arthritis**						
ICD-9	714.*	715.*				
ICD-10	M05-M06*	M15-M19*				
**Neuropathic Pain**						
ICD-9	350.*	353.*	354.*	355.*	356.*	53.12
	53.13	337.2*	250.6*			
ICD-10	G50.*	G51.*	G52.*	G53.*	G54.0-G54.6	G54.8-G59.*
	G60.*	B02.22; B02.23	B02.29	G90.5x; E13.4*		

NON-PHARMACOLOGICAL THERPY CPT CODES
All procedures codes for non-pharmacologic therapies for chronic non-cancer pain located at:
https://github.com/sbandar2/CNCP_Trt_Codes/blob/main/ProcedureCodes.xlsx

Note: Some codes sourced from: Faciszewski, Tom, Ron Jensen, and Richard L. Berg. "Procedural coding of spinal surgeries (CPT-4 versus ICD-9-CM) and decisions regarding standards: a multicenter study." *Spine* 28.5 (2003): 502-507. Lurie, Jon D., et al. "Indications for spine surgery: validation of an administrative coding algorithm to classify degenerative diagnoses." Spine 39.9 (2014): 769. Salt, Elizabeth, et al. "A description and comparison of treatments for low back pain in the US." Orthopedic nursing 35.4 (2016): 214. Arshi, Armin, et al. "Outpatient total knee arthroplasty is associated with higher risk of perioperative complications." JBJS 99.23 (2017): 1978-1986. Potts, Aaron, et al. "Practice patterns for arthroscopy of osteoarthritis of the knee in the United States." The American journal of sports medicine 40.6 (2012): 1247-1251. Yuan, Frank, et al. "Evidence-based practice in the surgical treatment of thumb carpometacarpal joint arthritis." The Journal of hand surgery 42.2 (2017): 104-112

NON-OPIOID PAIN MEDICATION NDC NUMBERS

All NDC codes for non-opioid pain medication treatment for chronic non-cancer pain located at:
https://github.com/sbandar2/CNCP_Trt_Codes/blob/main/nonopioidrx_final.csv
