# Supplementary material for: Trends in opioid and non-opioid treatment for chronic non-cancer pain and cancer pain among privately insured adults in the United States, 2012–2019
Source: PLoS One. 2022 Aug 10;17(8):e0272142. doi: 10.1371/journal.pone.0272142 (PMC9365134; doi:10.1371/journal.pone.0272142)
Supplement: S2 Appendix — (PDF) [file pone.0272142.s002.pdf]

S2 Appendix. Yearly Results

S2 Table1. Characteristics of individuals with chronic non-cancer pain (CNCP), 2012-2019

**Characteristic**	**Year**							
	**2012** (1,717,340)	**2013** (1,954,936)	**2014** (2,118,048)	**2015** (2,106,260)	**2016** (2,166,533)	**2017** (2,220,248)	**2018** (2,107, 622)	**2019** (1,556,737)
Mean Age	46	47	46	46	47	46	46	46
Female, % (n)	62 (1,072,140)	62 (1,213,145)	62 (1,317,999)	62 (1,299,489)	62 (1,339,234)	62 (1,366,130)	61 (1,294,432)	61 (954,234)
Region, % (n)								
Northeast	13 (223,665)	18 (348,232)	17 (364,126)	18 (380,445)	18 (391,365)	18 (408,322)	18 (388,099)	13 (202,227)
North Central	23 (392,697)	22 (429,140)	22 (471,111)	23 (477,342)	22 (480,513)	24 (532,645)	25 (523,447)	26 (396,943)
South	41 (710,601)	39 (769,409)	45 (954,411)	43 (914,002)	43 (938,950)	43 (948,481)	42 (885,124)	47 (734,646)
West	23 (387,545)	20 (398,241)	15 (321,783)	16 (329,892)	16 (346,216)	15 (326,517)	15 (306,804)	14 (218,669)
Unknown	< 1 (2,832)	1 (9,914)	< 1 (6,617)	< 1 (4,579)	< 1 (9,489)	< 1 (4,283)	< 1 (4,148)	< 1 (4,252)
CNCP Diagnosis, % (n)								
Low Back Pain	54 (926,564)	54 (1,060,496)	56 (1,180,171)	57 (1,199,016)	58 (1,262,389)	58 (1,284,311)	58 (1,216,812)	58 (895,649)
Serious Headache	29 (491,935)	29 (565,447)	30 (632,822)	30 (635,509)	30 (652,372)	31 (678,735)	31 (650,610)	31 (483,516)
Arthritis	28 (480,408)	28 (556,761)	28 (597,470)	28 (597,834)	29 (627,998)	29 (641,568)	29 (609,954)	29 (453,026)
Neuropathic Pain	15 (265,330)	16 (309,606)	16 (339,403)	16 (332,027)	12 (269,878)	12 (271,975)	12 (255,331)	12 (184,077)
> 1 CNCP Diagnosis, % (n)	23 (388,687)	24 (463,147)	25 (538,945)	26 (557,926)	26 (553,269)	25 (561,700)	25 (534,942)	25 (393,295)

S2Table2. Characteristics of individuals with cancer, 2012-2019

**Characteristic**	**Year**							
	**2012** (8,509)	**2013** (8,683)	**2014** (7,710)	**2015** (8,516)	**2016** (13,687)	**2017** (13,518)	**2018** (12,045)	**2019** (8,515)
Mean Age	51	52	52	52	53	53	53	53
Female, % (n)	65 (5,515)	63 (5,434)	61 (4,732)	55 (4,717)	48 (6,582)	47 (6,397)	48 (5,736)	48 (4,048)
Region, % (n)								
Northeast	17 (1,404)	20 (1,776)	21 (1,643)	22 (1,886)	22 (3,040)	21 (2,904)	22 (2,597)	17 (1,447)
North Central	24 (2,020)	22 (1,939)	22 (1,685)	22 (1,871)	22 (3,033)	24 (3,187)	25 (3,018)	24 (2,039)
South	38 (3,218)	37 (3,215)	42 (3,228)	41 (3,463)	40 (5,513)	39 (5,332)	38 (4,563)	45 (3,809)
West	22 (1,852)	20 (1,699)	15 (1,134)	15 (1,277)	15 (2,020)	15 (2,067)	15 (1,841)	14 (1,186)
Unknown	< 1 (15)	1 (54)	< 1 (20)	< 1 (19)	1 (81)	<1 (28)	<1 (26)	<1 (34)

S2Table3. Adjusted opioid prescription characteristics among individuals with chronic non-cancer pain who received an opioid prescription, 2012-2019

	Year							
	**2012**	**2013**	**2014**	**2015**	**2016**	**2017**	**2018**	**2019**
Number of opioid prescriptions per person, mean [95%CI]	5.2<br>[5.2, 5.3]	5.1*<br>[5.1, 5.1]	4.9*<br>[4.9, 4.9]	4.7*<br>[4.7, 4,7]	4.5*<br>[4.5, 4,5]	4.4*<br>[4.3, 4.4]	4.1*<br>[4.1, 4.1]	3.9*^<br>[3.9, 3.9]
MME/day among opioid prescriptions per person, mean [95%CI]	49.9<br>[49.7, 50.1]	50.0<br>[49.9, 50.2]	53.5*<br>[53.3, 53.6]	50.1*<br>[49.9, 50.2]	46.5*<br>[46.4, 46.6]	43.2*<br>[43.0, 43.3]	40.0*<br>[39.9, 40.1]	38.0*^<br>[37.9, 38.1]
Number of days per year with opioid prescriptions person, mean [95%CI]	74.2<br>[74.0, 74.5]	74.7*<br>[74.5, 74.9]	73.1*<br>[72.9, 73.3]	72.3*<br>[72.1, 72.5]	70.0*<br>[69.8, 70.2]	68.5*<br>[68.3, 68.8]	64.4*<br>[64.1, 64.6]	59.6*^<br>[59.3, 59.9]
Percentage of individuals w/ a prescription…								
>90 MME/day [95%CI]	13.9<br>[13.9, 14.0]	13.2*<br>[13.1, 13.2]	13.3*<br>[13.2, 13.4]	12.1*<br>[12.1, 12.2]	10.7*<br>[10.7, 10.8]	8.9*<br>[8.9,8.9]	6.6*<br>[6.6, 6.6]	4.9*^<br>[4.9, 5.0]
>200 MME/day [95%CI]	2.5<br>[2.5, 2.5]	2.5*<br>[2.5, 2.6]	3.4*<br>[3.4, 3.5]	2.6*<br>[2.6, 2.7]	1.8*<br>[1.8, 1.9]	1.3*<br>[1.2, 1.3]	0.9*<br>[0.8, 0.9]	0.8*^<br>[0.8, 0.9]
>7 Days Supply [95%CI]	56.3<br>[56.2, 56.4]	56.5*<br>[56.4, 56.6]	56.0*<br>[55.9, 56.1]	55.3*<br>[55.2, 55.4]	53.2*<br>[53.1, 53.3]	50.2*<br>[50.1, 50.3]	37.4*<br>[37.3, 37.6]	30.7*^<br>[30.6, 30.9]
>30 Days Supply [95%CI]	3.6<br>[3.6, 3.7]	3.2*<br>[3.2, 3.3]	2.9*<br>[2.8, 2.9]	2.0*<br>[1.9, 2.0]	1.7*<br>[1.6, 1.7]	1.5*<br>[1.4, 1.5]	1.2*<br>[1.1, 1.2]	0.9*^<br>[0.9, 0.9]

Note: * Wald test for differences w/ year prior $p < 0.05$; ^ Wald test for differences between 2012 and 2019 $p < 0.05$

S2Table4. Adjusted opioid prescription characteristics among individuals with cancer who received an opioid prescription, 2012-2019

	Year							
	**2012**	**2013**	**2014**	**2015**	**2016**	**2017**	**2018**	**2019**
Number of opioid prescriptions per person, mean [95%CI]	4.0 [3.9, 4.1]	3.9 [3.8, 4.0]	3.7* [3.6, 3.8]	3.5* [3.4, 3.6]	3.1* [3.0, 3.2]	2.9* [2.9, 3.0]	2.9 [2.8, 2.9]	2.7*^ [2.6, 2.8]
MME/day among opioid prescriptions per person, mean [95%CI]	62.4 [60.9, 63.8]	62.7 [61.2, 64.2]	66.1* [64.0, 68.1]	61.7* [60.1, 63.2]	59.2* [57.9, 60.4]	53.2* [52.2, 54.2]	47.3* [46.4, 48.2]	44.7*^ [43.3, 46.1]
Number of days per year with opioid prescriptions person, mean [95%CI]	34.8 [33.4, 36.1]	35.2 [33.9, 36.5]	33.9 [32.5, 35.2]	32.1 [30.8, 33.3]	28.1* [27.2, 29.1]	25.9* [25.0, 26.9]	24.2* [23.2, 25.2]	22.3*^ [21.1, 23.5]
Percentage of individuals w/ a prescription…								
>90 MME/day [95%CI]	26.2 [25.2, 27.2]	24.2* [23.3, 25.2]	23.0 [21.9, 24.0]	20.9* [20.0, 21.8]	17.3* [16.6, 18.0]	13.4* [12.8, 14.0]	10.1* [9.4, 10.6]	7.6*^ [7.0, 8.3]
>200 MME/day [95%CI]	3.6 [3.2, 4.1]	3.4 [3.0, 3.8]	4.1* [3.6, 4.5]	3.3* [2.9, 3.7]	2.8 [2.5, 3.1]	1.9* [1.6, 2.1]	1.5* [1.2, 1.7]	1.4^ [1.1, 1.7]
>7 Days Supply [95%CI]	47.5 [46.3, 48.6]	48.6 [47.4, 49.7]	50.4* [49.2, 51.6]	49.9 [48.7, 51.0]	44.0* [43.0, 44.8]	39.6* [38.7, 40.5]	27.9* [26.9, 28.7]	22.7*^ [21.8, 23.7]
>30 Days Supply [95%CI]	1.2 [0.9, 1.4]	1.1 [0.9, 1.4]	0.9 [0.7, 1.2]	0.7 [0.5, 0.9]	0.5 [0.4, 0.7]	0.5 [0.3, 0.6]	0.3 [0.2, 0.4]	0.3^ [0.2, 0.4]

Note: * Wald test for differences w/ year prior p <0.05; ^ Wald test for differences between 2012 and 2019 p <0.05
