# Supplementary material for: Trends in opioid and non-opioid treatment for chronic non-cancer pain and cancer pain among privately insured adults in the United States, 2012–2019
Source: PLoS One. 2022 Aug 10;17(8):e0272142. doi: 10.1371/journal.pone.0272142 (PMC9365134; doi:10.1371/journal.pone.0272142)
Supplement: S3 Appendix — (PDF) [file pone.0272142.s003.pdf]

S3 Appendix. Non-Opioid Medication and Non-Pharmacologic Procedure Types

S3Table1. Types of Non Opioid Prescriptions Among Individuals with Chronic Non-Cancer Pain

Non-opioid prescription category	Year							
	2012	2013	2014	2015	2016	2017	2018	2019
**INDIVIDUALS WITH CHRONIC NON-CANCER PAIN**								
5HT1 Agonists	4.2	4.3	4.4	4.4	4.4	4.5	4.8	4.9
Anticonvulsants	15.8	16.2	16.2	16.4	16.2	16.3	16.3	15.9
Local Anesthetic	0.0	0.0	0.0	0.0	0.0	0.0	0.0	0.0
NSAIDS	16.7	16.8	16.8	16.6	16.8	17.1	17.6	17.7
Other	20.6	20.4	20.4	20.2	20.2	19.8	18.7	18.4
Antidepressants	23.6	23.3	23.3	23.7	23.6	23.4	23.3	23.5
SNRI	6.7	6.5	6.5	6.8	6.7	6.6	6.6	6.7
SSRI	13.7	13.6	13.6	13.8	13.8	13.8	13.8	14.0
Tricyclic Antidepressants	3.2	3.2	3.2	3.1	3.1	3.0	2.9	2.8
Skeletal Muscle Relaxants	11.6	11.3	11.3	10.9	10.9	10.9	10.7	10.7
Steroids	7.4	7.6	7.6	7.7	7.9	8.1	8.5	9.0
**INDIVIDUALS WITH CANCER**								
5HT1 Agonists	0.9	1.1	0.7	0.6	0.5	0.6	0.7	0.5
Anticonvulsants	13.8	14.1	14.3	15.9	17.1	16.9	18.7	19.5
Local Anesthetic	0.0	0.0	0.0	0.0	0.0	0.0	0.0	0.0
NSAIDS	7.9	7.9	7.3	8.2	8.2	9.0	9.3	9.4
Other	31.8	31.7	31.2	32.1	31.9	31.5	30.0	28.4
Antidepressants	22.0	22.3	23.4	20.0	19.0	18.9	17.9	17.7
SNRI	5.7	5.5	5.9	4.6	4.4	4.2	4.3	4.0
SSRI	15.0	15.0	16.0	14.1	13.6	13.5	12.5	12.4
Tricyclic Antidepressants	1.3	1.8	1.5	1.3	1.0	1.2	1.1	1.3
Skeletal Muscle Relaxants	5.3	5.5	5.5	5.2	4.4	4.2	4.3	5.1
Steroids	18.4	17.4	17.6	18.1	18.9	18.8	19.2	19.4

S3Table2. Types of Non-Pharmacologic CNCP Therapy Among Individuals with Chronic Non-Cancer Pain

Non-pharmacologic therapy category	Year							
	2012	2013	2014	2015	2016	2017	2018	2019
Surgical	36.4	35.2	33.3	32.2	32.2	31.3	31.0	30.8
Minimally Invasive	22.4	22.5	22.4	22.5	22.6	23.8	23.8	23.9
Non-Invasive	42.4	44.4	46.0	47.1	48.4	48.5	49.6	50.9
