# Supplementary material for: Trends in opioid and non-opioid treatment for chronic non-cancer pain and cancer pain among privately insured adults in the United States, 2012–2019
Source: PLoS One. 2022 Aug 10;17(8):e0272142. doi: 10.1371/journal.pone.0272142 (PMC9365134; doi:10.1371/journal.pone.0272142)
Supplement: S4 Appendix — (PDF) [file pone.0272142.s004.pdf]

S4 Appendix. Unadjusted Results

S4Table 1. Unadjusted trends in receipt of opioid and non-opioid treatments among individuals with chronic non-cancer pain (CNCP) or cancer, 2012-2019

	2012	2013	2014	2015	2016	2017	2018	2019
**CNCP No Cancer**								
Opioid Prescription	50.0	47.8	45.2	43.5	41.2	37.7	33.3	31.0
Non-Opioid Prescription	66.6	66.4	66.0	66.0	66.3	65.9	65.8	66.9
Non-Pharmacologic Therapy	62.5	63.3	63.2	63.8	64.8	64.7	65.4	65.8
**Cancer and No CNCP**								
Opioid Prescription	86.4	86.0	84.5	86.0	85.1	82.3	80.0	78.7
Non-Opioid Prescription	75.4	74.6	74.2	75.2	72.9	74.2	75.9	78.6

S4Table 2. Unadjusted opioid prescription characteristics among individuals with chronic non-cancer pain who received an opioid prescription, 2012-2019

	2012	2013	2014	2015	2016	2017	2018	2019
Number of opioid prescriptions per person, mean [95%CI]	5.2	5.1	4.9	4.7	4.5	4.4	4.2	3.9
MME/day among opioid prescriptions per person, mean [95%CI]	50.0	50.1	53.3	50.0	46.5	43.2	40.1	37.9
Number of days per year with opioid prescriptions person, mean [95%CI]	73.5	74.0	72.7	71.9	70.2	69.0	65.2	61.2
Percentage of individuals w/ a prescription…								
>90 MME/day [95%CI]	14.1	13.2	13.2	12.1	10.8	8.9	6.6	5.0
>200 MME/day [95%CI]	2.5	2.5	3.4	2.6	1.9	1.3	0.9	0.8
>7 Days Supply [95%CI]	55.8	56.0	55.8	55.1	53.4	50.5	38.0	31.5
>30 Days Supply [95%CI]	3.6	3.2	2.8	1.9	1.7	1.5	1.2	0.9

S4Table 3. Unadjusted opioid prescription characteristics among individuals with cancer who received an opioid prescription, 2012-2019

	2012	2013	2014	2015	2016	2017	2018	2019
Number of opioid prescriptions per person, mean [95%CI]	3.5	3.4	3.1	3.0	2.6	2.4	2.3	2.2
MME/day among opioid prescriptions per person, mean [95%CI]	62.2	62.5	65.8	61.6	59.2	53.4	47.6	44.6
Number of days per year with opioid prescriptions person, mean [95%CI]	34.3	34.8	33.5	31.9	28.4	26.2	24.4	22.8
Percentage of individuals w/ a prescription…								
>90 MME/day [95%CI]	26.7	24.4	22.9	20.8	17.1	13.4	10.1	7.6
>200 MME/day [95%CI]	3.6	3.3	4.0	3.3	2.8	1.9	1.5	1.4
>7 Days Supply [95%CI]	47.5	48.4	50.4	49.9	44.0	39.6	27.8	23.1
>30 Days Supply [95%CI]	1.2	1.1	0.9	0.7	0.5	0.5	0.3	0.3
