# Supplementary material for: Trends in opioid and non-opioid treatment for chronic non-cancer pain and cancer pain among privately insured adults in the United States, 2012–2019
Source: PLoS One. 2022 Aug 10;17(8):e0272142. doi: 10.1371/journal.pone.0272142 (PMC9365134; doi:10.1371/journal.pone.0272142)
Supplement: S5 Appendix — (PDF) [file pone.0272142.s005.pdf]

S5 Appendix. Results for Sample with Chronic Non-Cancer Pain and Cancer

S5Table 1. Characteristics of individuals with cancer and chronic non-cancer pain (CNCP), 2012-2019

**Characteristic**	**Year**							
	**2012** (3,302)	**2013** (3,668)	**2014** (3,999)	**2015** (5,070)	**2016** (7,293)	**2017** (7,327)	**2018** (6,655)	**2019** (4,816)
Mean Age	52	52	52	52	53	53	53	52
Female, % (n)	67 (2,222)	66 (2,436)	65 (2,601)	60 (3,022)	55 (3,987)	54 (3,920)	54 (3,566)	55 (2,654)
Region, % (n)								
Northeast	15 (496)	19 (688)	21 (822)	20 (1,020)	21 (1,544)	21 (1,528)	21 (1,409)	15 (737)
North Central	24 (783)	23 (858)	22 (886)	23 (1,167)	22 (1,638)	24 (1,761)	25 (1,662)	27 (1,293)
South	39 (1,285)	38 (1,383)	43 (1,718)	43 (2,171)	41 (3,023)	41 (3,039)	40 (2,648)	45 (2,165)
West	22 (736)	20 (727)	14 (560)	14 (702)	14 (1,051)	13 (984)	14 (916)	13 (604)
Unknown	< 1 (2)	<1 (12)	< 1 (13)	< 1 (10)	1 (37)	<1 (15)	<1 (20)	<1 (17)
CNCP Diagnosis, % (n)								
Low Back Pain	47 (1,564)	48 (1,767)	49 (1,948)	52 (2,630)	57 (4,168)	56 (4,099)	54 (3,600)	55 (2,671)
Serious Headache	38 (1,263)	39 (1,439)	38 (1,533)	38 (1,940)	37 (2,704)	38 (2,764)	38 (2,527)	38 (1,837)
Arthritis	25 (835)	25 (921)	26 (1,027)	27 (1,355)	29 (2,149)	30 (2,186)	30 (2,009)	30 (1,457)
Neuropathic Pain	23 (747)	25 (919)	27 (1,068)	24 (1,225)	16 (1,150)	16 (1,157)	15 (1,025)	15 (737)
> 1 CNCP Diagnosis, % (n)	28 (935)	32 (1,163)	33 (1,319)	34 (1,716)	33 (2,412)	33 (2,440)	32 (2,105)	33 (1,577)

S5Figure1. Adjusted opioid and non-opioid treatments among individuals with chronic non-cancer pain (CNCP) and

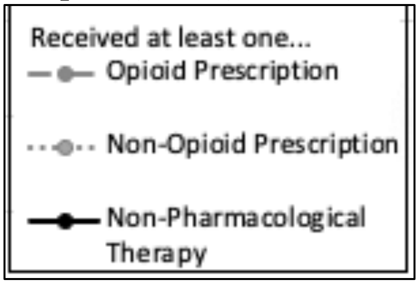

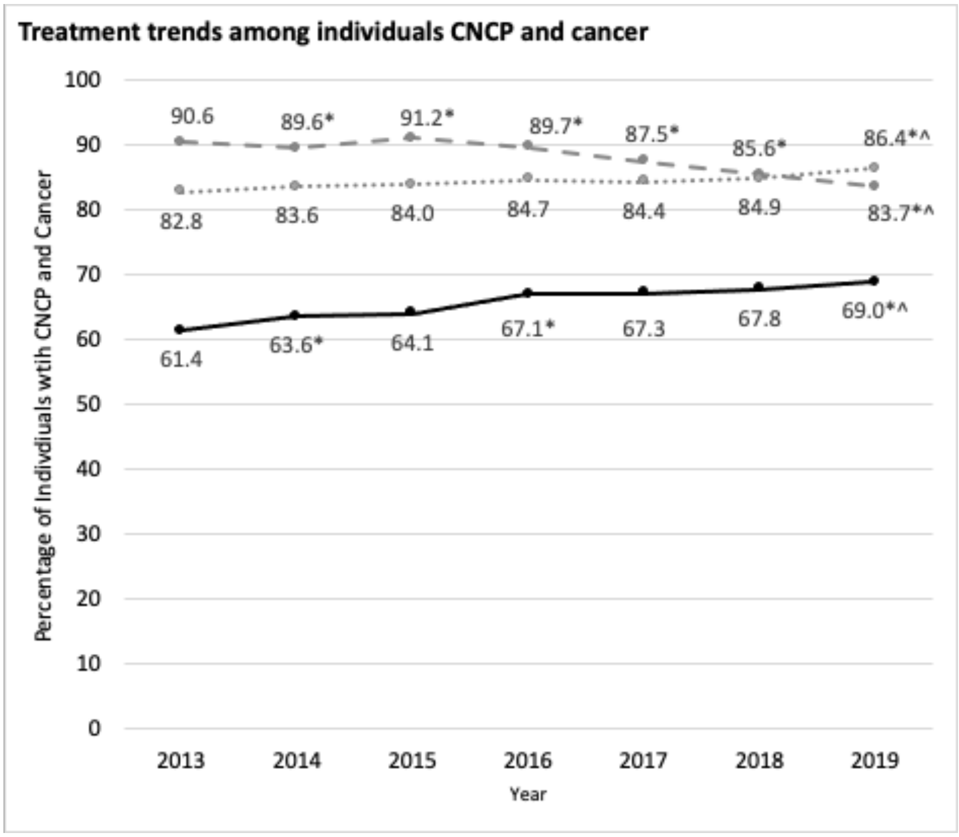

NOTE: * Wald test for differences w/ year prior p <0.05; ^ Wald test for differences between 2012 and 2019 p <0.05

S5Table 2. Adjusted opioid prescription characteristics among individuals with cancer and chronic non-cancer pain who received an opioid prescription, 2012-2019

	Year							
	**2012**	**2013**	**2014**	**2015**	**2016**	**2017**	**2018**	**2019**
Number of opioid prescriptions per person, mean [95%CI]	6.4 [6.2, 6.7]	6.4 [6.1, 6.6]	6.1 [5.9, 6.3]	5.8* [5.6, 6.0]	5.4* [5.3, 5.6]	4.9* [4.8, 5.1]	4.7* [4.6, 4.9]	4.6^ [4.4, 4.8]
MME/day among opioid prescriptions per person, mean [95%CI]	69.1 [66.1, 72.1]	70.9 [67.9, 74.0]	73.3 [70.1, 76.6]	74.5 [71.4, 77.5]	64.8* [62.7, 66.7]	59.1* [57.2, 60.8]	54.7* [52.9, 56.4]	49.9*^ [47.9, 51.8]
Number of days per year with opioid prescriptions person, mean [95%CI]	69.4 [66.1, 72.7]	70.7 [67.5, 73.8]	68.4 [65.3, 71.4]	65.5 [62.9, 68.2]	63.3 [61.1, 65.4]	57.4* [55.3, 59.5]	52.2* [49.9, 54.5]	50.4^ [47.7, 53.0]
Percentage of individuals w/ a prescription…								
>90 MME/day [95%CI]	32.3 [30.6, 34.0]	30.9 [29.4, 32.5]	30.0 [28.5, 31.5]	28.4 [27.1, 29.7]	24.6* [23.6, 25.6]	20.6* [19.6, 21.6]	15.5* [14.6, 16.4]	12.6*^ [11.6, 13.6]
>200 MME/day [95%CI]	6.2 [5.4, 7.1]	7.2 [6.3, 8.1]	7.1 [6.3, 8.0]	7.2 [6.4, 7.9]	4.9* [4.4, 5.4]	3.6* [3.2, 4.1]	3.0* [2.5, 3.4]	2.6^ [2.1, 3.1]
>7 Days Supply [95%CI]	66.7 [65.0, 68.4]	65.9 [64.3, 67.5]	67.4 [65.8, 68.9]	66.9 [65.6, 68.3]	63.8* [62.6, 65.0]	58.1* [56.9, 59.3]	44.5* [43.2, 45.8]	38.8*^ [37.3, 40.3]
>30 Days Supply [95%CI]	2.9 [2.3, 3.5]	2.6 [2.1, 3.2]	2.5 [2.0, 3.0]	1.8* [1.4, 2.2]	1.5 [1.2, 1.7]	0.9* [0.7, 1.1]	0.8 [0.5, 1.0]	0.4^ [0.3, 0.7]

Note: * Wald test for differences w/ year prior $p < 0.05$; ^ Wald test for differences between 2012 and 2019 $p < 0.05$
